# Meiotic Recombination Analyses of Individual Chromosomes in Male Domestic Pigs (*Sus scrofa domestica*)

**DOI:** 10.1371/journal.pone.0099123

**Published:** 2014-06-11

**Authors:** Nicolas Mary, Harmonie Barasc, Stéphane Ferchaud, Yvon Billon, Frédéric Meslier, David Robelin, Anne Calgaro, Anne-Marie Loustau-Dudez, Nathalie Bonnet, Martine Yerle, Hervé Acloque, Alain Ducos, Alain Pinton

**Affiliations:** 1 INRA, UMR1388 Génétique, Physiologie et Systèmes d’Elevage, Castanet-Tolosan, France; 2 Université de Toulouse INPT ENSAT, UMR1388 Génétique, Physiologie et Systèmes d’Elevage, Castanet-Tolosan, France; 3 Université de Toulouse INPT ENVT, UMR1388 Génétique, Physiologie et Systèmes d’Elevage, Toulouse, France; 4 UE1372 GenESI Génétique, Expérimentation et Système Innovants, Surgères, France; University of Science and Technology of China, China

## Abstract

For the first time in the domestic pig, meiotic recombination along the 18 porcine autosomes was directly studied by immunolocalization of MLH1 protein. In total, 7,848 synaptonemal complexes from 436 spermatocytes were analyzed, and 13,969 recombination sites were mapped. Individual chromosomes for 113 of the 436 cells (representing 2,034 synaptonemal complexes) were identified by immunostaining and fluorescence *in situ* hybridization (FISH). The average total length of autosomal synaptonemal complexes per cell was 190.3 µm, with 32.0 recombination sites (crossovers), on average, per cell. The number of crossovers and the lengths of the autosomal synaptonemal complexes showed significant intra- (i.e. between cells) and inter-individual variations. The distributions of recombination sites within each chromosomal category were similar: crossovers in metacentric and submetacentric chromosomes were concentrated in the telomeric regions of the p- and q-arms, whereas two hotspots were located near the centromere and in the telomeric region of acrocentrics. Lack of MLH1 foci was mainly observed in the smaller chromosomes, particularly chromosome 18 (SSC18) and the sex chromosomes. All autosomes displayed positive interference, with a large variability between the chromosomes.

## Introduction

During meiosis, recombination between homologous chromosomes generates two kinds of recombination products: crossovers (CO) and non-crossovers (NCO). NCO result in the unidirectional transfer of short genomic segments (gene conversion) and therefore have a limited impact on genetic diversity. Conversely, CO result in the reciprocal exchange of large chromosome segments between homologues and play a major role in the genetic variability of populations. CO are also necessary for the correct segregation of chromosomes during meiosis-I [1]. Lack of CO can result in chromosomal non-disjunction, leading to the production of aneuploid gametes [2]. In the most severe cases, low levels of CO can be associated with impaired spermatogenesis [3], [4].

Recombination sites are not distributed homogeneously along the chromosomes. Indeed, two COs very rarely occur near to one another. This phenomenon, known since 1916, has been termed “interference” [5] and different models have been proposed to explain it [6], [7]. Moreover some chromosomal regions, known as recombination hotspots [8], are preferentially affected by recombination. In Humans, 23,000 crossover hotspots, 1–2 kb in length and spaced approximately every 50–100 kb, have been identified [9]–[11]. They exhibit different recombination activities and are located in genic as well as in intergenic regions. Recently, the PR domain zinc finger protein 9 (PRDM9) has been shown to play a major role in the specification of such recombination hotspots in mice, humans and pigs [12], [13]. PRDM9 encodes a histone methyl transferase that allows trimethylation of the H3K4 histone. Active hot spots in mice are enriched for H3K4me3 [14]. Moreover, the DNA sequence matching the predicted PRDM9 binding site is present in 40% of the hot spots identified by linkage disequilibrium [15].

Historically, meiotic recombination studies have relied on the physical localization of chiasmatas [16], or on linkage analysis [17]. The discovery of proteins involved in CO formation (especially MLH1 and MLH3 observed in late recombination nodules) allowed the direct study of recombination using immunocytological approaches [18]. Such approaches have also been used to analyse CO interference, for example by fitting the distribution of inter-CO distances to the gamma model [19]. Until now, immunocytological techniques have been used to study recombination patterns in various mammalian species such as mouse [20], Man [21], cattle [22], cat [23], shrew [24], mink [25], and dog [26], as well as 3 species of primates [27], but not in pigs.

The domestic pig karyotype (2n = 38) is composed of 2 sex chromosomes and 18 pairs of autosomes (5 metacentrics, 7 submetacentrics and 6 acrocentrics) [28]. This karyotypic structure is relatively similar to that of Humans. For this reason, the pig species is a much better animal model for meiotic recombination studies than other mammalian species, like dogs or mice, whose karyotypes contain only acrocentric chromosomes.

In this paper, for the first time in the domestic pig, we present unique data on the meiotic recombination of males with normal karyotypes, obtained by direct immunocytological approach. Distribution of the MLH1 foci and the estimated strength of interference on the 18 porcine autosomal pairs were determined by identifying each autosome by multiple specific loci *in situ* hybridization.

## Materials and Methods

### Ethics Statement

Our experiments were conducted in accordance with the European Directive 2010/63/EU on the protection of animals used for scientific purposes, and validated by the Ethic Committee for Animal Experimentation of the Poitou Charentes region (France) (N°CE2012-2). The experimentation agreement number for the experimental farm in which the animals were raised was A-17-661.

### Biological Material

Testicular samples were collected by surgical hemi-castration of 4 boars of different genotype (Large White, Meishan, minipig and crossbred between Large White and Creole) and age (195, 360, 695 and 307 days, respectively). Classical cytogenetic analyses (GTG banding karyotyping) were carried out according to Ducos et al. (1998) [29]. Histopathological analyses were carried out as described by Barasc et al. (2014) [30]. Analyses of the boar karyotypes and gonads did not reveal any alteration. Moreover, the seminal parameters of the boars (concentration, mobility, and morphological parameters) were within the normal limits.

### Immunocytology

Meiotic cells were prepared as previously described by Massip et al. (2010) [31]. The synaptonemal complex proteins 3 (SCP3) and 1 (SCP1), MutL homolog 1 protein (MLH1) and centromeres were detected using the following primary antibodies: rabbit anti-SCP3 (1∶1000; ABCAM, Cambridge, UK), rabbit anti-SCP1 (2∶1000; ABCAM, Cambridge, UK), mouse anti-MLH1 (2∶100; Becton Dickinson, Francklin Lakes, NJ), Human anti-kinetochore (1∶100; Antibodysuits Incorporated, Davis), respectively, and prepared in a solution of PBT (PBS +0.16% BSA +0.1% Tween). The secondary antibodies consisted of Alexa 594 conjugated donkey anti-rabbit (1∶100, Molecular Probes), Alexa 488 conjugated goat anti-mouse (1∶100, Molecular Probes), and AMCA conjugated donkey anti-human (1∶100, Jackson, Grove, PA, USA). Spermatocytes were captured and analyzed using the Cytovision FISH imaging system (Leica Microsystems, Nanterre, France).

### Fluorescent *In Situ* Hybridization (FISH)

**Figure 1:**
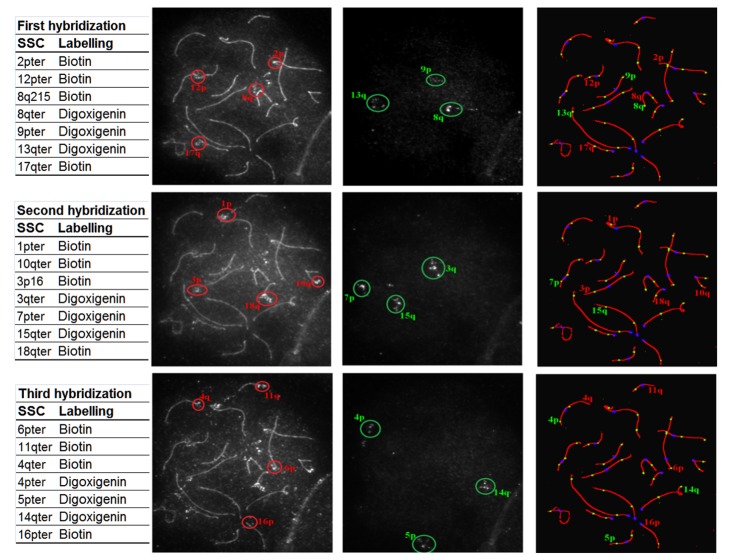
Example of identification of the 18 pig autosomes. 1st column: set of 7 BACs used for each hybridization with their positions and labeling nucleotide. 2nd column: raw image of the capture of the 4 BACs labeled with biotin and revealed in red. 3rd column: raw image of the capture of the 3 BACs labeled with digoxigenin and revealed in green. 4th column: identification of chromosome arms on spermatocyte after immunolocalization of SCP3 (red), MLH1 (green) and kinetochores (blue).

FISH experiments were performed on the same slides, according to Massip et al. (2010) [31], with slight modifications. The 18 autosomal bivalents were identified by 3 successive hybridizations of BAC probe combinations, as presented in Figure 1. These BAC probes were selected in the telomeric regions of the chromosomes [32] and were obtained from the Biological Resources Center-GADIE (http://www-crb.jouy.inra.fr/) [33]. Probes were labeled with biotin or digoxygenin using the bioprime labeling system (Invitrogen) and revealed using Alexa 594 conjugated to Streptavidin (Molecular Probes, Eugene, OR, USA) and FITC conjugated mouse anti-digoxygenin antibodies (Sigma, St Louis, MO).

### Image and Statistical Analyses

After identification of each bivalent, the images were analyzed to determine the length of the synaptonemal complexes (SC), and the positions of the centromere and CO, using MicroMeasure 3.3 software [34]. The relative positions of the MLH1 signals and centromeres were expressed as percentages of the total SC lengths, starting from the end of the q arms. The distances between adjacent MLH1 foci (relative distances expressed as % of total SC length) were used to estimate the interference for each chromosome. The strength of interference was estimated by fitting the frequency distribution of the inter-CO distances to the gamma model, as explained in Broman and Weber (2000) [35] and Lian et al (2008) [19]. Maximum likelihood estimates of the υ parameter of the gamma function were obtained using the free online Wessa software [36].

Differences in the length of SC and the number of MLH1 foci per cell, between the 4 boars, were examined by one-way analyses of variance (ANOVA) and subsequent multiple comparison analyses (Tukey test). The same tests were used to compare the relative lengths between mitotic and meiotic chromosomes. Differences in the number of MLH1 foci per chromosome between two individuals were examined by Student’s *t-*test. Pearson correlation analyses were used to investigate the relationships between the number of MLH1 foci and the length of autosomal SCs per cell and per chromosome, the relationships between the two sets of distances from the centromere to the nearest COs on the p- and q-arms, and the relationships between the meiotic *vs* mitotic chromosome length differences and the percentage of GC content.

## Results and Discussion

### Analyses of Synaptonemal Complex Lengths

It has been demonstrated that the physical SC lengths are continuously varying along the pachytene substages [37], [38]. Therefore, knowledge of the physical (absolute) SC lengths *per se* is of relatively limited interest. However, frequencies of recombination are frequently expressed in the literature in CO/µm. Consequently, such results are also given and discussed below.

In this study, immunocytological techniques were used to analyze 436 pachytene cells from 4 normal boars of different ages and breeds. The average total autosomal SC lengths per cell for these 4 boars are presented in Table 1. High intra-individual variability was observed, (the largest for the Meishan boar, ranging from 146 µm to 268 µm). Nevertheless, the between-boar differences for this trait were highly significant (P<0.001). The difference between the two extremes (179.6 µm for Mini 1 and 199.3 µm for Meishan, respectively) represented 10% of the overall mean for this trait (190.3 µm). This value is slightly higher than the 161.5 µm already published for swine [39]. However, this latter study was conducted on a small number of spermatocytes, using electron microscopy and, as previously mentioned, high intra-individual variability could explain this difference.

**Table 1:** Number of spermatocytes analyzed, mean number of MLH1 foci, mean SC length per cell, and percent of SC without MLH1 foci on autosome or XY pairs for each individual.

				Total autosomal SC length per cell (µm)		MLH1 foci per cell		% of SC without MLH1 foci	
Individuals	Genetic type	No. of cells analyzed	FISH*	Mean ±SD	Range	Mean ±SD	Range	autosomes	Sex chromosomes
LW 1.0	Large White	148	46	192.79^b^ ±16.73	160.72; 237.15	30.19^a^ ±3.38	21; 32	2.8	27
Mini 1	Minipig	88	---	179.63^a^ ±19.52	142.55; 230.75	37.33^b^ ±4.44	24; 46	1.2	26
Meish	Meishan	102	67	199.29^c^ ±22.37	146.23; 268.97	30.80^a^ ±3.57	21; 40	2.3	32
LWxCr	Large White X Creole	98	---	186.99^ab^ ±20.46	142.45; 248.41	31.37^a^ ±3.33	24; 40	2.3	39
	**Total**	**436**	**113**						

* number of spermatocytes analyzed by FISH (all 18 autosomes identified).

a, b and ^c^ superscripts: when a superscript letter is common to two experimental values, these two experimental values are not statistically different (P>0.05).

### Comparison between Mitotic and Meiotic Chromosome Lengths

The relative lengths of mitotic and meiotic chromosomes (identified by FISH) were compared for the LW1.0 and Meish boars (Table 2). Some SC were significantly longer than the corresponding mitotic chromosomes (SSC3, SSC6 and SSC14; P<0.001), whereas others were significantly shorter (SSC1, SSC4, SSC5, SSC8, SSC9, and SSC11; P<0.001). Nevertheless, the correlation between the two measures was very high for both individuals (r = 0.953 for LW1.0 and r = 0.952 for Meish). Similarly, for some chromosomes, the positions of the centromeres between the SC and mitotic chromosomes differed significantly (Table 2). These results demonstrated that, for a given SC, the variation in length (between the mitotic chromosome and SC) on a chromosome arm was independent of the variation in length on the other arm. Similar results were obtained in Humans and mice [20], [40]. Differences in GC content and gene density between chromosomes have been proposed to explain this phenomenon.

**Table 2:** Comparisons of the relative lengths and centromere index between synaptonemal complexes and mitotic metaphasic chromosomes.

SSC	Synaptonemal complexes^a^								Mitotic chromosomes^b^			
	LW 1.0				Meish							
	Relative length^c^ (%)		Centromere index^d^ (%)		Relative length^c^ (%)		Centromere index^d^ (%)		Relative length^c^ (%)		Centromere index^d^ (%)	
	mean	SD	mean	SD	Mean	SD	mean	SD	mean	SD	mean	SD
1	10.70***	0.94	31.47	3.00	10.50***	0.99	30.80	2.91	11.98	0.74	32.19	4.97
2	6.60	0.54	35.01	4.12	6.50*	0.65	35.25	3.35	6.76	0.39	33.88	5.78
3	6.81***	0.85	42.49***	3.87	7.44***	0.92	48.16***	4.17	5.95	0.33	35.79	6.81
4	5.25*	0.55	38.26**	3.25	5.13***	0.54	36.24	3.28	5.57	0.49	34.94	5.47
5	4.35***	0.30	46.64***	3.43	4.33***	0.42	47.46***	2.95	4.72	0.33	37.10	7.35
6	8.09***	1.14	23.68*	3.14	8.09***	1.23	24.01*	4.02	7.32	0.43	26.10	6.19
7	5.37	0.36	24.31***	2.56	5.51	0.59	22.29***	3.53	5.57	0.41	32.03	7.86
8	5.26***	0.46	45.22***	3.68	5.31***	0.66	46.05***	3.81	5.97	0.41	37.17	7.23
9	5.25***	0.43	46.90***	3.65	5.03***	0.56	47.42***	3.47	5.79	0.42	33.14	6.78
10	4.28	0.49	49.13***	5.56	4.32	0.43	47.45***	4.04	4.32	0.35	40.77	7.47
11	3.18***	0.32	47.85***	3.21	2.92***	0.31	46.26***	2.60	3.49	0.32	39.56	6.88
12	3.51**	0.33	48.36***	3.19	3.42	0.42	47.46***	4.11	3.26	0.26	42.39	6.12
13	8.80	1.21			9.11**	1.25			8.49	0.58		
14	8.14***	1.18			8.10***	1.18			6.18	0.46		
15	5.79	0.56			5.49**	0.70			5.83	0.51		
16	3.28**	0.36			3.37*	0.42			3.54	0.34		
17	2.93**	0.32			2.86*	0.33			2.72	0.34		
18	2.42	0.27			2.55	0.27			2.53	0.40		

a Average values for 828 SCs from LW 1.0 and 1206 SCs from Meish.

b Average values for 50 metaphases from representative animals.

c Percent of total autosomal SC length.

d Centromere index = SC length for p arm/(SC length for q+p arms).

* P<0.05; ** P<0.01; *** P<0.001 compared to mitotic chromosomes.

### Number of MLH1 Foci Per Cell

The number of MLH1 foci per spermatocyte was estimated by analysing 436 meiocytes from the 4 boars (Table 1). The overall distribution was Gaussian with an average number of 32.0±4.5 MLH1 foci per cell.

Considerable intra-individual variability (between cells) was again observed in the number of MLH1 foci per spermatocyte (from 21 to 40 MLH1 foci per cell for the Meishan boar, for example). Large differences were also observed between individuals. Nevertheless, only the value obtained for the minipig boar (Mini 1) differed significantly from the others. Similar results had already been obtained in Humans [40]–[42], mice [43] and in former pig studies based on genetic (linkage) analyses [13], [44]. In the pig study by Vingborg et al. (2009) [44], the estimated total recombination length was 1,441 cM (centi Morgan, genetic map unit), i.e. 18% lower than the 1,760 cM found in the study by Tortereau et al. (2012) [13]. Considering that one recombination event corresponds to 50 cM, the total recombination length estimated in our study ranged from 1,509 cM to 1,866 cM (for LW 1.0 and Mini 1, respectively). These results are consistent with those of Vingborg *et al.* (2009) [44] and Tortereau *et al.* (2012) [13].

### Number of MLH1 Foci Per Autosome

By combining immunostaining and FISH experiments, we were able to determine the number of MLH1 foci per individual chromosome for two animals (LW1.0 and Meish; Table 3). One hundred and thirteen cells representing 2034 bivalents were analyzed. As expected, the average number of MLH1 signals was higher for the large chromosomes than for the small ones. Comparisons between the two boars did not reveal any difference in the number of MLH1 foci per chromosome (P>0.05) except for SSC 15. These results suggest that, for 2 individuals with a comparable total number of MLH1 foci per cell, the number of foci per chromosome remains identical.

**Table 3:** Absolute lengths and numbers of MLH1 foci for individual autosomal SCs.

SSC	Individuals	Absolute SC length (µm)		MLH1 foci			Genetic length (cM)^a^	Physical length (Mb)^b^	Recombination rate	
		mean	SD	mean	SE	SD			cM per Mb	CO per µm
1	LW1.0	20.59	2.43	2.72	0.10	0.66	135.9	315.3	0.43	0.132
	Meish	21.63	3.55	2.67	0.09	0.70	133.6		0.42	0.124
2	LW1.0	12.71	1.53	1.98	0.07	0.45	98.9	162.6	0.61	0.156
	Meish	13.37	2.10	1.82	0.06	0.49	91.0		0.56	0.136
3	LW1.0	13.12	2.12	1.96	0.09	0.63	97.8	144.8	0.68	0.149
	Meish	15.32	2.72	1.93	0.07	0.61	96.3		0.67	0.126
4	LW1.0	10.07	1.08	1.78	0.06	0.42	89.1	143.5	0.62	0.177
	Meish	10.54	1.66	1.78	0.07	0.55	90.2		0.63	0.168
5	LW1.0	8.36	0.88	1.74	0.08	0.57	90.9	111.5	0.82	0.208
	Meish	8.87	0.98	1.61	0.07	0.58	83.1		0.75	0.182
6	LW1.0	15.57	2.58	2.09	0.09	0.63	104.3	157.8	0.66	0.134
	Meish	16.62	3.16	2.07	0.08	0.64	103.7		0.66	0.125
7	LW1.0	10.31	0.90	1.91	0.09	0.59	95.7	134.8	0.71	0.185
	Meish	11.32	1.73	2.07	0.08	0.66	103.7		0.77	0.183
8	LW1.0	10.11	1.18	1.76	0.06	0.43	88.0	148.5	0.59	0.174
	Meish	10.88	1.66	1.79	0.06	0.51	89.6		0.60	0.165
9	LW1.0	10.09	1.02	1.80	0.08	0.54	92.2	153.7	0.60	0.179
	Meish	10.31	1.41	1.87	0.05	0.42	93.3		0.61	0.181
10	LW1.0	8.24	1.25	1.57	0.10	0.65	85.7	79.1	1.08	0.190
	Meish	8.84	1.15	1.64	0.06	0.51	83.3		1.05	0.186
11	LW1.0	6.11	0.69	1.30	0.09	0.63	69.8	87.7	0.80	0.213
	Meish	5.98	0.70	1.18	0.06	0.52	62.7		0.72	0.197
12	LW1.0	6.75	0.72	1.28	0.08	0.54	67.0	63.6	1.05	0.190
	Meish	6.99	0.97	1.30	0.07	0.60	70.2		1.10	0.186
13	LW1.0	16.94	2.91	2.17	0.09	0.64	108.7	218.6	0.50	0.128
	Meish	18.76	3.51	2.10	0.09	0.70	106.8		0.49	0.112
14	LW1.0	15.71	2.95	1.87	0.07	0.45	93.5	153.9	0.61	0.119
	Meish	16.67	3.22	1.97	0.07	0.55	98.5		0.64	0.118
15	LW1.0	11.17	1.70	1.70	0.08	0.51	86.7	157.7	0.55	0.152
	Meish	11.33	2.19	1.90	0.06	0.46	96.2		0.61	0.167
16	LW1.0	6.29	0.69	1.30	0.07	0.47	65.2	86.9	0.75	0.207
	Meish	6.90	1.05	1.22	0.07	0.55	65.1		0.75	0.177
17	LW1.0	5.63	0.82	1.13	0.06	0.40	57.8	69.7	0.83	0.201
	Meish	5.86	0.77	1.07	0.05	0.40	56.3		0.81	0.183
18	LW1.0	4.64	0.48	0.89	0.06	0.38	51.3	61.2	0.84	0.192
	Meish	5.23	0.70	0.90	0.05	0.39	51.7		0.84	0.171

a Genetic length = number of MLH1 foci x 50 (in centi-morgan. cM).

b Physical length (in megabase. Mb). Data obtained from the porcine sequence 10.2 (http://www.ncbi.nlm.nih.gov/genome/84?project_id=28993).

### Lack of MLH1 Foci on Some Bivalents

The lack of MLH1 signals on some bivalents (autosome or sex bivalent) was noted (Table 1). Sex bivalents never presented more than one MLH1 signal, and an absence of MLH1 signal was particularly frequent (from 26 to 39%) on this particular bivalent as compared with the other chromosomes (from 1.2 to 2.8%). Comparable results were obtained in human studies, which also revealed great variability between individuals (9 to 44% of sex chromosomes without MLH1 foci [45]). It has been suggested that the sex bivalent XY is predisposed to nondisjunction due to a frequent lack of recombination in the pseudoautosomal region [46], [47]. This is consistent with the higher rates of aneuploidy estimated for sex chromosomes as compared with autosomes [48]. However, the proportions of XY bivalents lacking MLH1 signals are much higher than the aneuploidy rates of sex chromosomes reported in Man and pigs (in sperm as well as in newborn [30], [49]–[51]). This suggests that a certain proportion of germ cells lacking the MLH1 signal on the XY bivalent are eliminated during spermatogenesis. On the other hand, these high rates of XY bivalents without any MLH1 signal observed in our study, as well as in previous human studies, could also be due, in part, to technical problems (limited accessibility of the MLH1 antibodies to this specific bivalent due to their particular shape and molecular environment, higher rate of hybridization failure due to the limited size of the pseudoautosomal region, …). This specific point has not been documented so far, and should be investigated further.

In contrast to results obtained in humans [52], we did not observe any obvious relationship between the frequency of spermatocytes lacking MLH1 signals in the XY bivalents and the total number of MLH1 signals per cell (Table 1). However, it is very difficult to document a relationship between two variables using only 4 points (individuals). Complementary data are required to document this point more thoroughly. We also compared, for each individual, the total numbers of autosomal MLH1 signals per cell: (a) in cells with one MLH1 signal on the XY bivalent, and (b) in cells without any MLH1 signal on the XY bivalent. The (a - b) difference was positive in all cases, but low and statistically different from zero for 2 boars only: (a–b) = 1.6 (P = 0.06) for LW1.0; (a–b) = 0.6 (P = 0.39) for LWxCr; (a–b) = 1.3 (P = 0.03) for Meish and (a–b) = 2.9 (P = 0.01) for Mini1.

The frequencies of autosomes without any MLH1 signal were much lower, and varied from 1.2% to 2.8% between individuals, as indicated previously (Table 1). These proportions are comparable to those observed in mice (4%, [18]), dog (0.5%, [26]) and shrew (0.7%, [24]). The lack of MLH1 signal mainly concerned the small chromosomes, with 87% of them being observed on SSC5, SSC10, SSC11, SSC12, SSC16, SSC17 and SSC18. The smallest pig autosome (SSC 18) aggregated on its own 29% of the cases. Similar results have been reported in humans [45], where the absence of MLH1 signal was most frequently observed for one of the smallest chromosomes (HSA 22).

### Relationship between the Number of MLH1 Foci and the SC Length

**Figure 2:**
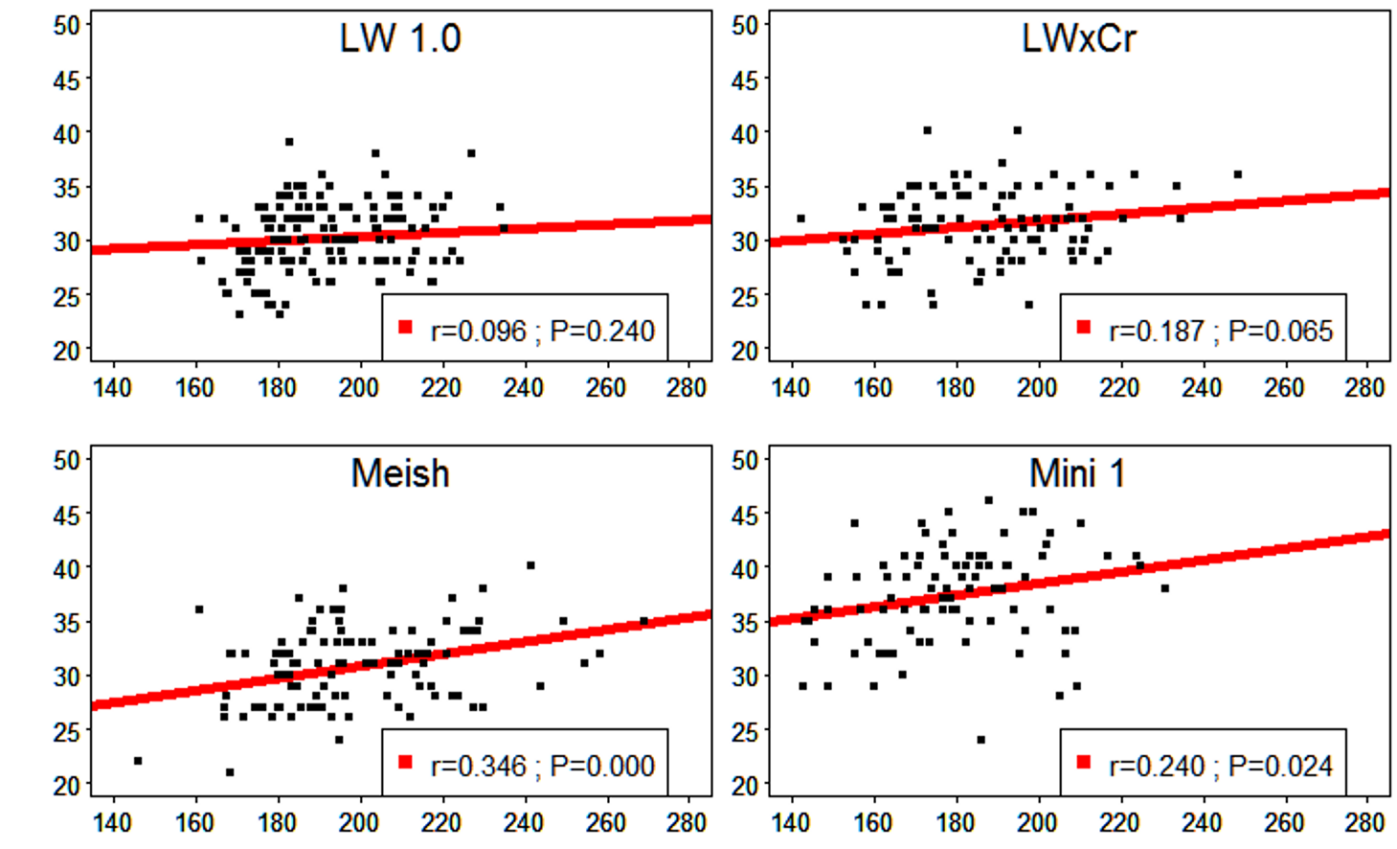
Relationship between the number of MLH1 foci and the total SC length for the four boars analyzed. x-axis: total length of autosomal SC per cell (µm); y-axis: total number of MLH1 foci per cell.

Relationships between the total number of MLH1 foci per cell and the total SC length per cell were analyzed for each individual (Figure 2). The two variables were positively, albeit (very) moderately, correlated (the correlation was positive but not statistically different from 0 for the LW1.0 boar). Comparable results have been obtained in human studies (correlation values ranging from 0.02 to 0.69 [53]).

In our study, the average total autosomal SC length per cell was 190.3 µm, for an average of 32.0 MLH1 foci per cell. This gives, on average, one MLH1 signal every 5.9 µm which is comparable to the results obtained in humans (1 focus/6.0 µm, [21]), dogs (1 focus/6.2 µm, [26]), mink (1focus/6.3 µm, [25]) and shrew (1 focus/6.5 µm, [24]) but quite different from those obtained for the cat (1foci/4.6 µm, [23]) and mouse (1 foci/7.1 µm, [20]). However, considerable variability was observed between individuals (which were of different genotypes), ranging from 4.8/µm for Mini 1 to 6.5/µm for Meish. Studies carried out in mice [43] suggested that the between-strain differences in crossover levels were associated with a variation in the length of the meiotic axes (smaller DNA loops and longer SCs in the strains exhibiting large numbers of MLH1 foci per cell). Our results (Table 1) do not support this hypothesis since boars with the lowest total SC lengths per cell (Mini1 and LWxCr) presented the highest rates of recombination (37.33 and 31.37 MLH1 foci per cell, respectively). Thus, the variability in the total number of MLH1 foci observed between the four boars cannot be fully explained by the variability in the total autosomal SC lengths, and vice versa. Another explanation for the between-boar difference would be that the overall genetic background could regulate SC length independently of CO formation. As mentioned in the introduction, allelic variation for PRDM9 has been associated with hotspot activity in mice and humans [12], [54], [55]. Variation of RNF212 alleles also affects genome-wide recombination in humans [56]–[58]. Such allelic variations could explain part of the inter-individual variation of the MLH1 foci number observed in our study. No data supporting this hypothesis could be found in the literature for the pig species. This should be investigated in the near future.

**Figure 3:**
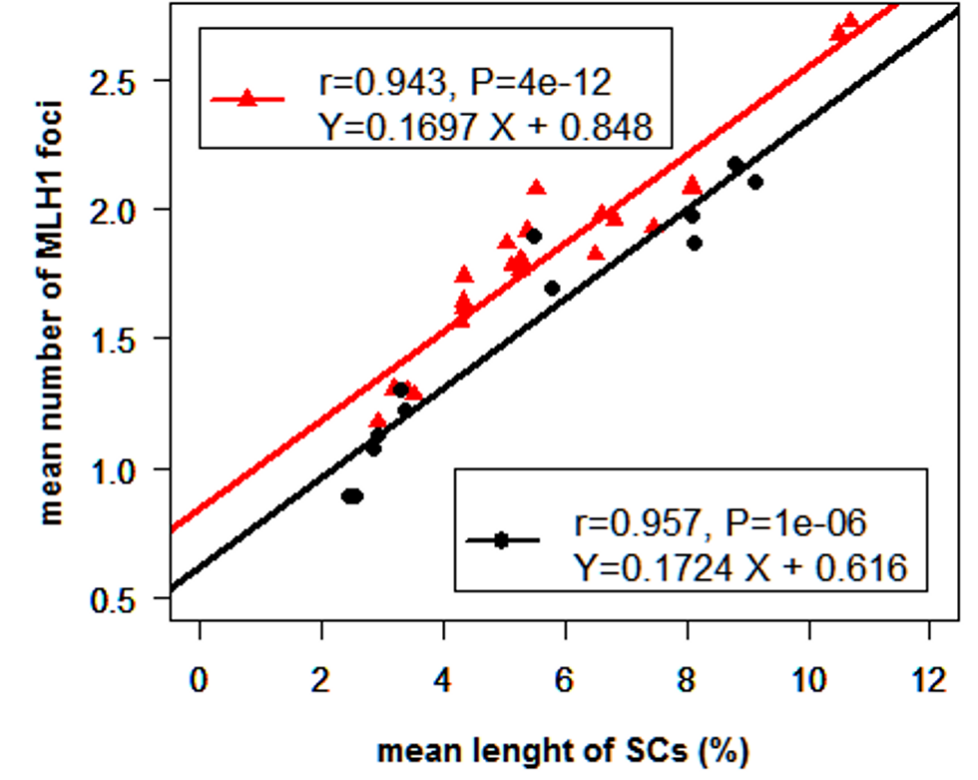
Relationship between the average number of MLH1 foci and the average absolute SC length for the 2 types of autosomes: non-acrocentrics (SSC1 to SSC12) in red and acrocentrics (SSC13 to SSC18) in black.

The relationships between the number of MLH1 foci and the absolute SC length (in µm) were also analyzed using the data obtained for each bivalent independently (Table 3). As expected, the two variables were highly correlated in the two studied animals (r = 0.936 and r = 0.912 for LW 1.0 and Meish, respectively). A similar relationship has already been observed in other mammals [21], [59]. In order to determine whether this relationship was comparable in acrocentric and non-acrocentric (i.e. metacentric and submetacentric) chromosomes, data from the two individuals (LW 1.0, and Meish) were pooled (to increase the number of observations per chromosome category). This pooling of data from two different individuals obliged us to consider relative SC lengths instead of absolute SC lengths in the subsequent analyses. The obtained correlations were slightly higher when acrocentric and non-acrocentric chromosomes were considered separately (r = 0.957 and r = 0.943, respectively) as compared with r = 0.924 when all the autosomes were considered simultaneously. As shown in Figure 3, the linear regression lines of the number of MLH1 foci (Y) on the length of SC (X) for the two kinds of chromosomes presented similar slopes (P = 0.89) but significantly different intercepts (higher for non-acrocentric; P<0.05). This indicates that, in general, metacentric and submetacentric chromosomes present higher recombination rates than acrocentrics. However, results obtained in other species indicate a different situation when the metacentric chromosome results from a fusion between 2 acrocentrics. Indeed, studies of individuals from different mice species or from bovidae differing only in the presence of Robertsonian translocations, revealed a reduction of recombination on the fused acrocentric chromosomes (which became metacentric), as compared to the free initial acrocentric chromosomes [22], [60]. Different Robertsonian translocations have been identified in several young boars controlled in our laboratory [61]. This should provide us with an opportunity to more thoroughly document this point in the near future.

### Distributions of the MLH1 Foci on Individual Chromosomes

**Figure 4:**
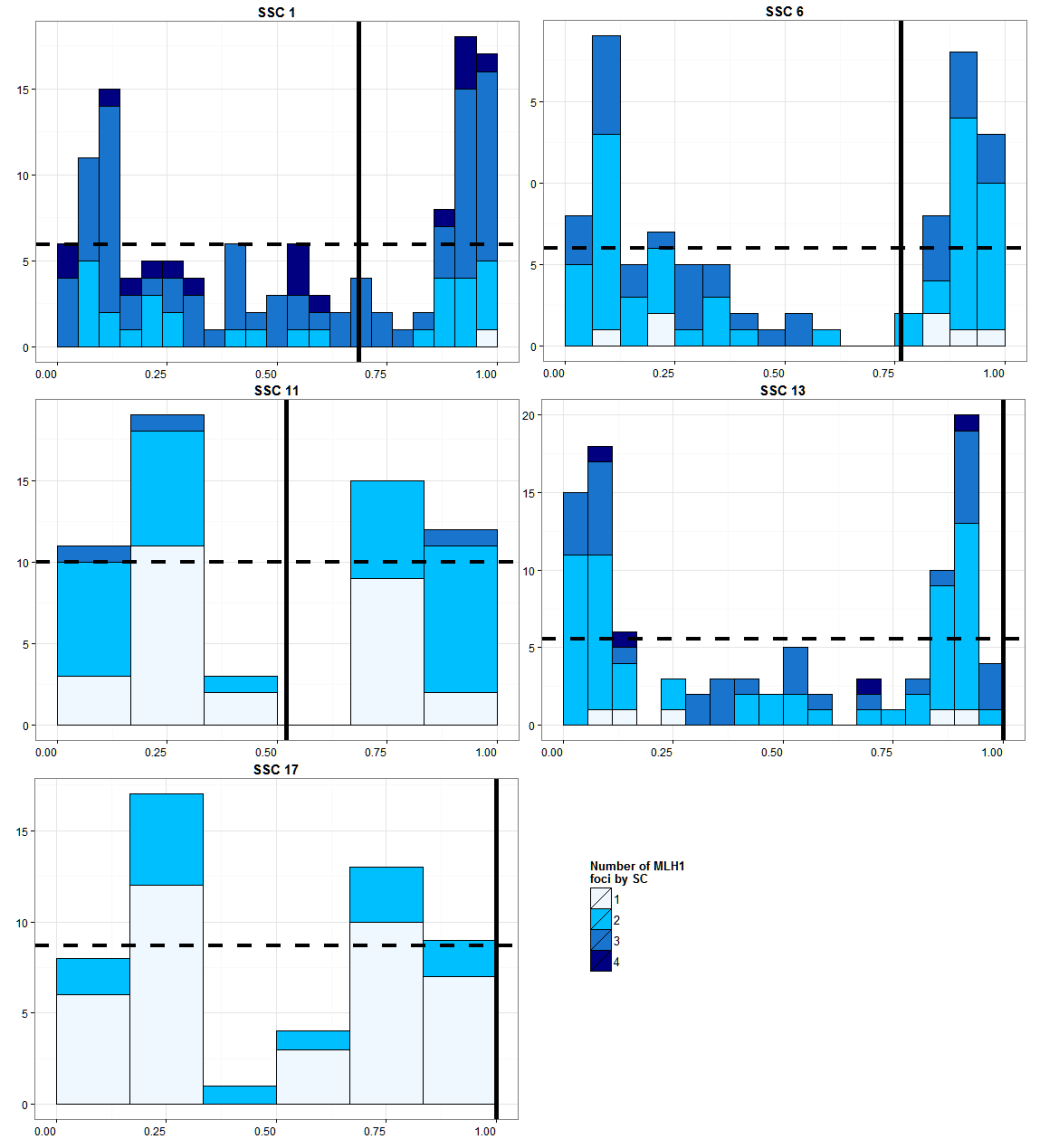
Distributions of MLH1 foci for 5 representative autosomes from LW 1.0. For each autosome, the x-axis indicates the position of the signals on the SC, from the q (left) arm to the p (right) arm. This axis is divided into a number of intervals proportional to the length of the SC. The y-axis indicates the number of MLH1 foci in each interval. The vertical line in bold represents the centromere and the dotted line the average number of MLH1 signals per SC. For each autosome, the columns (from lighter to darker blue) indicate bivalent with 1, 2, 3 or 4 MLH1 foci.

The distributions of MLH1 foci for some representative chromosomes of LW1.0 are presented in Figure 4, and for the 18 autosomes of the two analyzed boars in Figure S1. Overall, the MLH1 foci distributions were not uniform. As already reported in the literature, the CO were concentrated in specific areas known as recombination hotspots. The two main hotspots for non-acrocentric chromosomes were located in telomeric regions, one on the p-arm, and the other on the q-arm. Conversely, the frequencies of MLH1 foci in the centromeric regions of non-acrocentric chromosomes were generally low (even an absence was noted for some chromosomes, SSC 11 for instance). Significantly different distributions were observed for acrocentrics. Two hotspots were frequently observed in this kind of chromosomes, as in non-acrocentrics, but one was located close to the centromeres. Similar recombination patterns were obtained in pigs by Tortereau et al. (2012) using high-density linkage map analysis [13]. These results seemed to indicate that, in acrocentric chromosomes, the centromere does not hinder the formation of CO in the porcine male, in contrast to other mammalian species like dogs [26]. For these latter, it was proposed that recombination in the centromeric regions might interfere with kinetochore assembly, and that a reduction of recombination in these regions could prevent this phenomenon. However, it has been shown that the swine DNA sequences in the centromeric regions differ between acrocentric and non-acrocentric chromosomes. More precisely, the acrocentric swine subgenome presents a higher degree of DNA sequence homogenization (nature of sequences and copy number) than the metacentric one [33], [62], [63]. This could provide an explanation for the difference observed between the two species.

**Figure 5:**
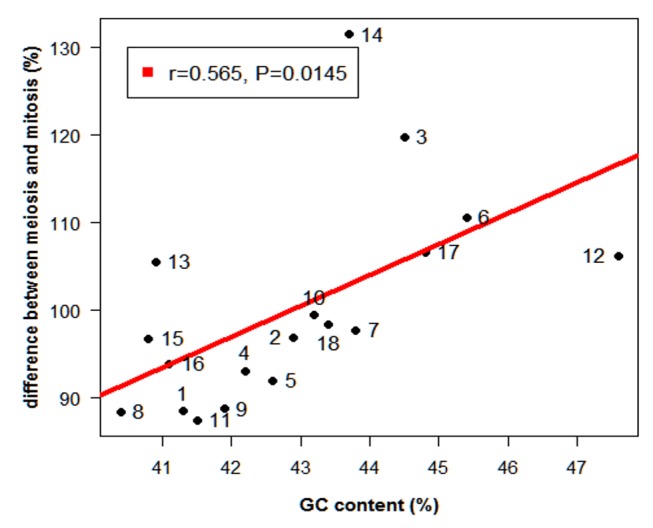
Relationship between the meiotic *vs* mitotic chromosome length differences (y-axis) and the percentage of GC content (x-axis). The difference in relative length between meiotic and mitotic chromosomes is expressed as a percentage (% difference = relative SC length/relative mitotic chromosome length × 100). GC content of the porcine chromosomes was obtained from the porcine sequence 10.2 (http://www.ncbi.nlm.nih.gov/genome/84?project_id=28993).

Positive correlations between sequence parameters (GC content, repetitive elements content and short sequence) and recombination rate have been reported in humans [64], mice [65], dogs [66] and pigs [13]. Considering that the relationship between the SC length and CO frequency is also positive (Figure 3), this would imply that chromosomes with high GC content would present longer SC than expected from the corresponding mitotic chromosomes, as well as high levels of recombination. Our results, presented in Figure 5, confirm these predictions. Indeed, SSC3, SSC6 and SSC14 have a high GC content and are longer than their corresponding mitotic chromosomes, in contrast to SSC1 and SSC8. Moreover, SSC3 and SSC6, for example, present higher levels of recombination than SSC 1 (0.68 cM/Mb, 0.66 cM/Mb and 0.42 cM/Mb respectively).

### Analysis of Interference

**Figure 6:**
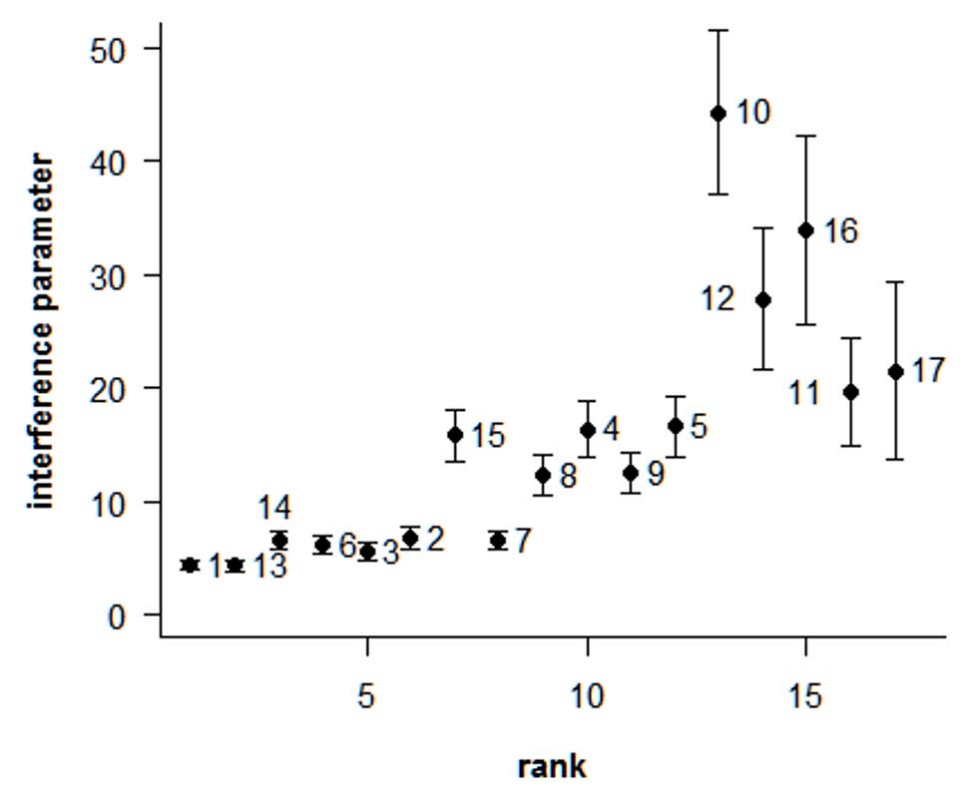
Relationship between the estimated υ parameter (±SD) and the mean SC length for each autosome. Data obtained from the pooled data of the 2 individuals studied using FISH. There is no value for SSC18 because this chromosome rarely presented more than one MLH1 signal.

The interference parameter for the different porcine autosomes was estimated from the pooled results of the 2 individuals analyzed (Figure 6). The MLH1 interfoci distances were expressed as the distances between two adjacent MLH1 foci in percentages of the SC lengths. As for numerous other organisms [19], [67], the gamma model provides a good fit to the inter-foci distribution for our recombination data. The estimated υ parameter, for all the chromosomes, was significantly greater than 1 (ranging from 4.2 for SSC1 to 44.3 for SSC10), which demonstrates a positive interference as observed in humans [19], or mice [68]. The level of interference differed significantly between chromosomes, the strength of interference being globally lower for large chromosomes than for small ones. This is consistent with results obtained in humans and mice [40], [69], [70], which indicated that the strength of interference is modulated by the SC length.

The average relative distance between adjacent MLH1 foci, in all bivalents presenting only two foci (n = 1099), was 67.5% of the bivalent length (range 7.5–92.8%). This result is very close to the values reported in humans (68%, [21]), and mice (70%, [20]).

### Effects of the Centromere on Interference

**Figure 7:**
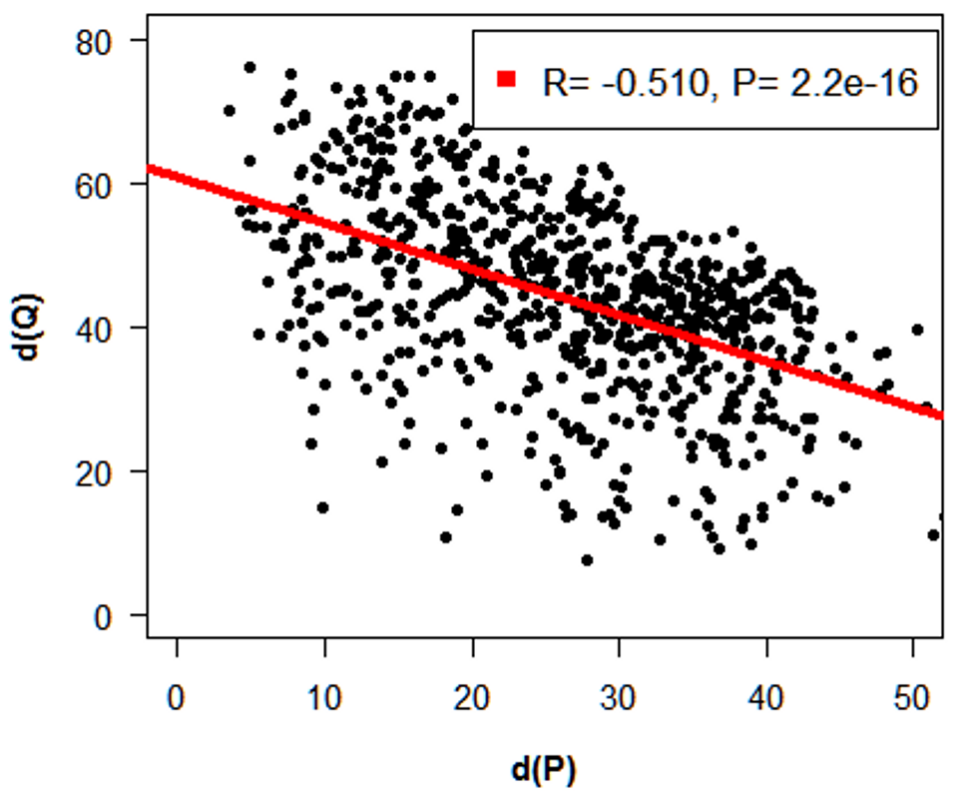
Relationship between the distances from the centromere to the nearest CO on the p [d(P)] (x-axis) and q [d(Q)] (y-axis) arms of chromosomes with at least one CO on each arm. The distances are expressed as percentages of the SC lengths. A significant negative correlation was found between the two distances, indicating that interference acts across the centromere.

Evidence from several species suggests that interference acts across the centromere [19], [35], [71]–[73]. To document this point, the relationship between (x-axis) the distances between the centromere and the nearest MLH1 foci measured on the p arm [d (P)], and (y-axis) the distances between the centromere and the nearest MLH1 foci on the q arm [d (Q)], was analyzed using data from SC with at least one MLH1 signal on each arm (Figure 7). The distances between the centromeres and the MLH1 foci were expressed as percentages of the SC lengths. The analysis revealed a significant negative correlation between the two distances (r = −0.510; P<0.0001), confirming that interference runs across the centromere in swine chromosomes.

## Conclusion

This article reports original and unique data concerning the direct analysis of meiotic recombination and interference in the pig species, a major agricultural as well as an interesting model species for chromosome research. The domestic pig is only the third species, after humans and mice, to have been analyzed by immunolocalization combined with chromosome specific *in situ* hybridization, which allowed a direct analysis of crossover frequency and distribution, as well as an estimation of the interference strength on each individual SC. Moreover, use of the immunolocalization approach made it possible to study recombination in the sexual chromosomes for the first time in pigs. Some important results already obtained in humans and/or mice have been confirmed, whereas others were more specific to pig chromosomes, as for instance the difference in recombination rates between acrocentric and non-acrocentric chromosomes (lower rate for acrocentrics). This work provides us with reference data concerning meiotic recombination and interference in normal pigs. Further work will be carried out in our group to i) produce comparable data for the female meiosis, and ii) document the impact of different kinds of chromosomal rearrangements on recombination and interference.

## Supporting Information

Figure S1 **Distribution of MLH1 foci for all autosomes from the LW 1.0 and Meish.** For each autosome, the x-axis indicates the position of the signals on the SC, from the q (left) arm to the p (right) arm. This axis is divided into a number of intervals proportional to the length of the SC. The Y-axis indicates the number of MLH1 foci in each interval. The vertical line in bold represents the centromere and the dotted line the average number of MLH1 signals per SC. For each autosome, the columns (from lighter to darker blue) indicate bivalent with 1, 2, 3 or 4 MLH1 foci.
(DOCX)
