# Supplementary material for: Meiotic Recombination Analyses of Individual Chromosomes in Male Domestic Pigs (Sus scrofa domestica)
Source: PLoS One. 2014 Jun 11;9(6):e99123. doi: 10.1371/journal.pone.0099123 (PMC4053413; doi:10.1371/journal.pone.0099123)
Supplement: Figure S1 — Distribution of MLH1 foci for all autosomes from the LW 1.0 and Meish. For each autosome, the x-axis indicates the position of the signals on the SC, from the q (left) arm to the p (right) arm. This axis is divided into a number of intervals proportional to the length of the SC. The Y-axis indicates the number of MLH1 foci in each interval. The vertical line in bold represents the centromere and the dotted line the average number of MLH1 signals per SC. For each autosome, the columns (from lighter to darker blue) indicate bivalent with 1, 2, 3 or 4 MLH1 foci. (DOCX) [file pone.0099123.s001.docx]

**Figure S1:** Distribution of MLH1 foci for all autosomes from the LW 1.0 and Meish.

- LW 1.0


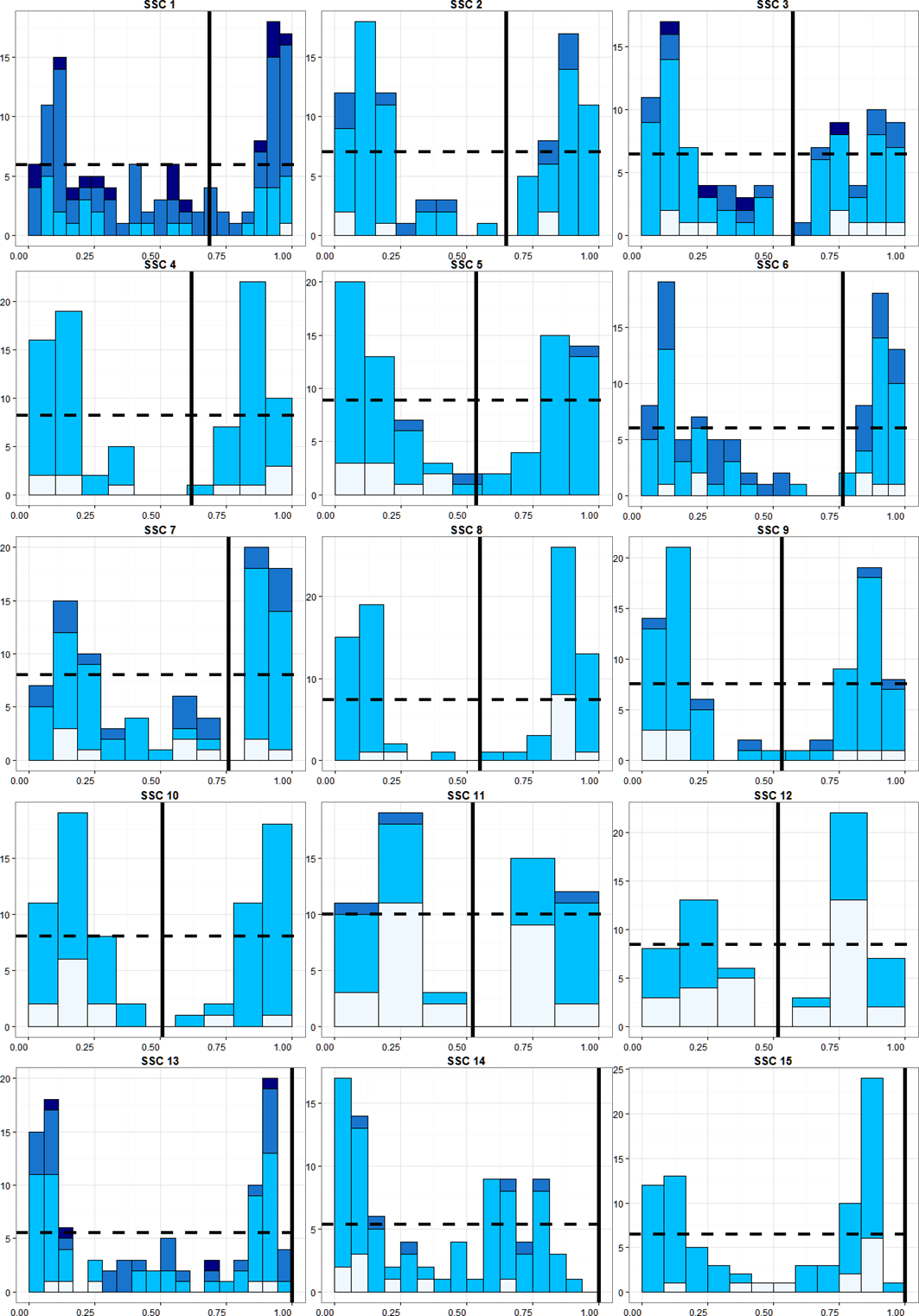


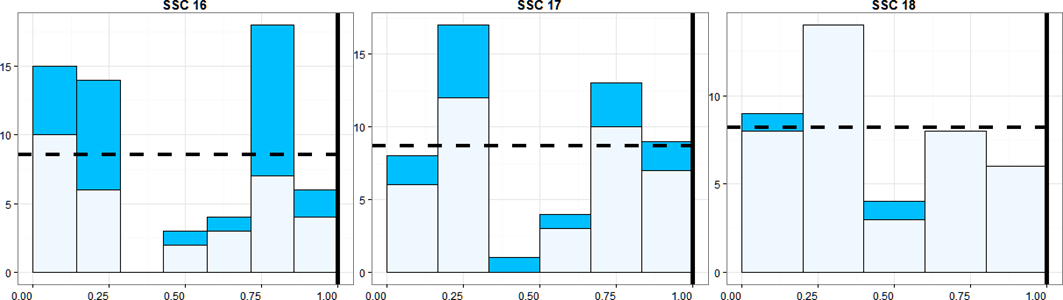


- Meish


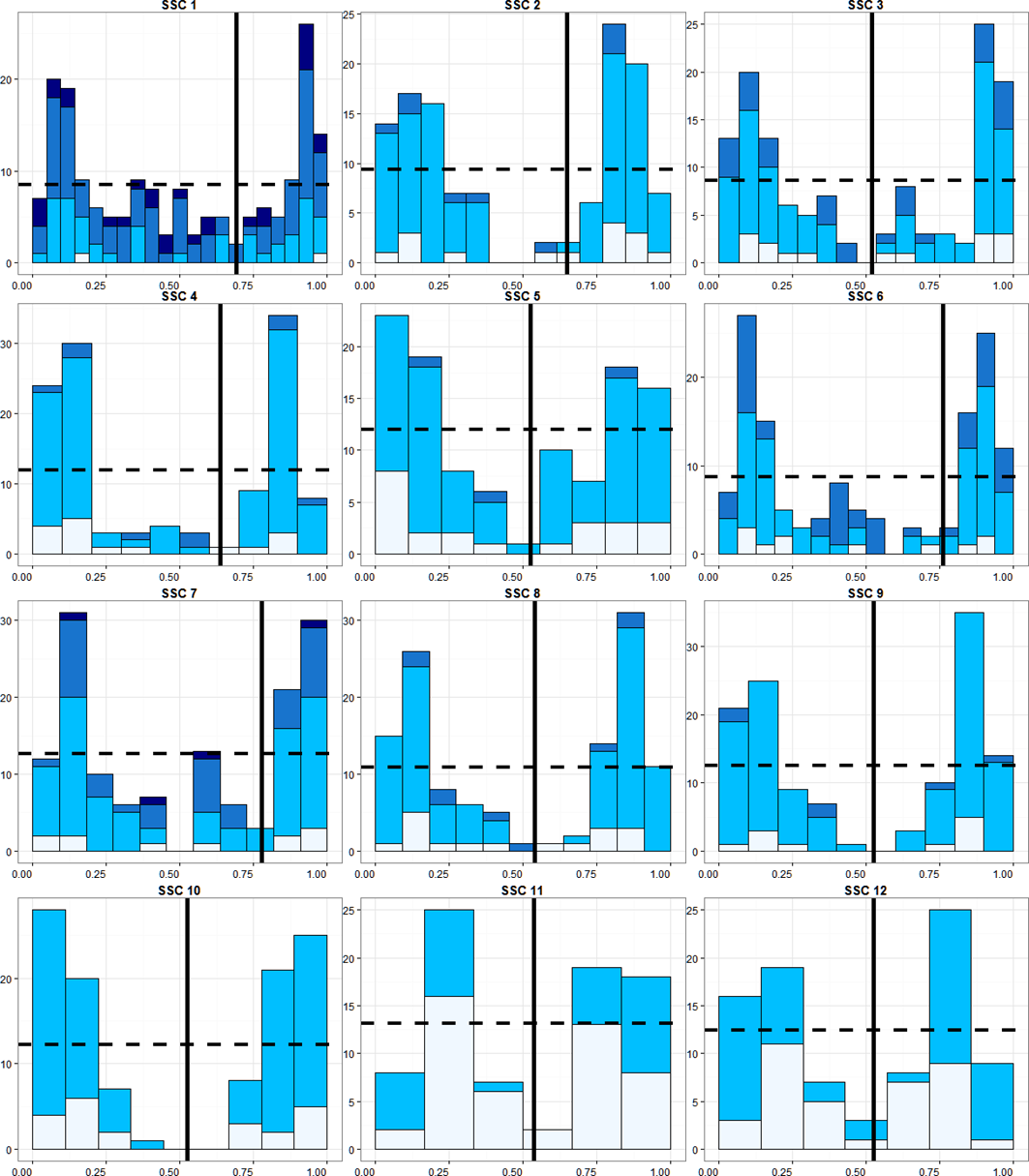


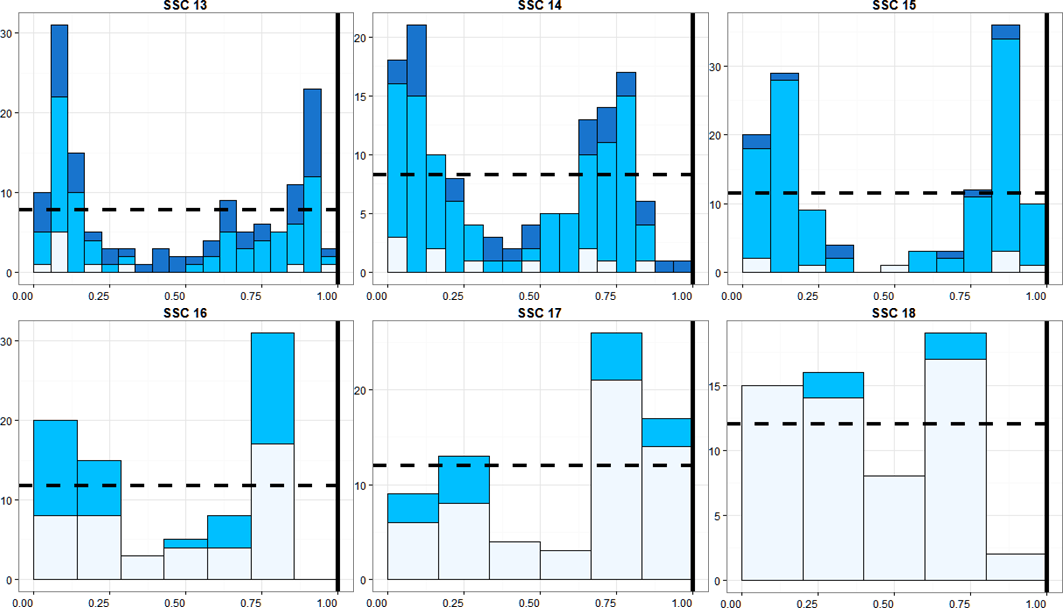


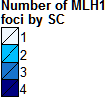


For each autosome, the x-axis indicates the position of the signals on the SC, from the q (left) arm to the p (right) arm. This axis is divided into a number of intervals proportional to the length of the SC. The Y-axis indicates the number of MLH1 foci in each interval. The vertical line in bold represents the centromere and the dotted line the average number of MLH1 signals per SC. For each autosome, the columns (from lighter to darker blue) indicate bivalent with 1, 2, 3 or 4 MLH1 foci.
